# Identification of Prognostic Genes in Leiomyosarcoma by Gene Co-Expression Network Analysis

**DOI:** 10.3389/fgene.2019.01408

**Published:** 2020-02-04

**Authors:** Jun Yang, Cuili Li, Jiaying Zhou, Xiaoquan Liu, Shaohua Wang

**Affiliations:** ^1^ Department of Pediatrics, The University of Hong Kong-Shenzhen Hospital, ShenZhen, China; ^2^ Department of Pediatrics, Women and Children Health Institute of FuTian, University of South China, ShenZhen, China

**Keywords:** leiomyosarcoma, prognosis, weighted gene co-expression network analysis (WGCNA), TCGA, recurrence

## Abstract

**Background/Aims:**

Leiomyosarcoma (LMS) is a tumor derived from malignant mesenchymal tissue associated with poor prognosis. Determining potential prognostic markers for LMS can provide clues for early diagnosis, recurrence, and treatment.

**Methods:**

RNA sequence data and clinical features of 103 LMS were obtained from the Cancer Genome Atlas (TCGA) database. Application Weighted Gene Co-Expression Network Analysis (WGCNA) was used to construct a free-scale gene co-expression network, to study the interrelationship between its potential modules and clinical features, and to identify hub genes in the module. The hub gene function was verified by an external database.

**Results:**

Twenty-four co-expression modules were constructed using WGCNA. A dark red co-expression module was found to be significantly associated with disease recurrence. Functional enrichment analysis and GEPIA and ONCOMINE database analyses demonstrated that hub genes CDK4, CCT2, and MGAT1 may play an important role in LMS recurrence.

**Conclusion:**

Our study constructed an LMS co-expressing gene module and identified prognostic markers for LMS recurrence detection and treatment.

## Introduction 

Leiomyosarcoma (LMS) is a highly malignant mesenchymal-derived tumor with varying degrees of smooth muscle differentiation, accounting for approximately 10% of soft tissue sarcomas (Noujaim et al., 2015; Pautier et al., 2015). These tumors occur mainly in adults in any body location and are associated with very high mortality. Leiomyosarcoma is divided into a variety of pathological subtypes according to cell morphology and molecular atypia, including typical leiomyosarcoma, epithelioid leiomyosarcoma, and pleomorphic leiomyosarcoma. Because this type of tumor is prone to recurrence and metastasis, it often has invasive clinical characteristics and poor prognosis. The 5-year recurrence rate is less than 40% (Serrano and George, 2013). Although many genes and signaling pathways have been identified to improve detection and treatment of LMS, surgical removal of tumors is currently the most effective way to treat leiomyosarcoma. Poor prognosis of LMS is related to a higher degree of malignancy, larger tumor volume, and deeper tumor site (Hayashi et al., 2010; Ognjanovic et al., 2012; Croce and Chibon, 2015). Therefore, identification of new biomarkers to assess malignancy and prognosis of LMS is essential.

Weighted correlation network analysis (WGCNA) is a systematic biological approach used to describe the pattern of gene association between different samples. WGCNA analysis uses correlation coefficient weights to make the connections between genes in the network obey scale-free networks, which is more biologically significant (Langfelder and Horvath, 2008). WGCNA can be used to identify highly synergistically altered gene sets and identify candidate biomarker genes or therapeutic targets based on the association of gene set connectivity and phenotype (Radulescu et al., 2018). Compared to genes that only focus on differential expression, WGCNA uses thousands of the most variable genes or all of the genes to identify the set of genes of interest and conducts a significant association analysis with the phenotype. WGCNA may make full use of information, and to convert thousands of genes and phenotypes into several gene sets and phenotypes, eliminating the need for multiple hypothesis testing (Zuo et al., 2018).

In this study, we constructed a co-expression network of LMS through WGCNA to systematically analyze the pathogenesis of LMS and tumorigenesis. Our goal is to study new and key biomarkers and to develop a better understanding of the molecular mechanisms of LMS to provide new strategies for diagnosis and treatment of diseases.

## Materials And Methods

### Data Collection

The mRNA sequence data and corresponding clinical traits of LMS were downloaded from the TCGA database (https://tcga-data.nci.nih.gov/tcga/), which contained 103 tumor tissues. Gene symbol annotation information was used to match probes with corresponding genes. TCGA was publicly available and in an open access platforms. As a result, ethics committee approval was not required.

### Co-Expression Network Construction With WGCNA and Target Prediction

The WGCNA algorithm runs in the R software package (http://www.r-project.org/) to assess the importance of genes and their associated modules by calculation the correlation coefficient between any two genes (Person Coefficient). To measure whether two genes have similar expression patterns, screening is performed and values above a pre-determined threshold are considered similar. WGCNA analysis uses the correlation coefficient weighting value, which is the N^th^ power of the gene correlation coefficient, so that the connections between the genes in the network obey the scale-free networks, which is more biologically significant. A hierarchical clustering tree was constructed based on the weighted correlation coefficients of genes. Genes were classified according to expression patterns, and genes with similar patterns were classified into one module. Different branches of the cluster tree represent different gene modules, and different colors represent different modules. This strategy allows for tens of thousands of genes can be divided into dozens of modules based on gene expression patterns, which is a process of extracting information. After weighted correlation analysis, we predicted target genes using a co-expression network produced using Cytoscape 3.7.0 software.

### Construct Module-Trait Relationships of LMS

Gene modules are linked to the traits of the study to screen for key gene modules. We used “module eigenvalues” to represent the combined value of the gene set expression of the module. Therefore, each module can be associated with a trait by the eigenvector of the module and the correlation coefficient of the phenotype or the saliency P value of the module. In addition, the modules do not exist in isolation, but are related to each other. Using a network heat map, the connections between the trait association module and other modules can be visualized.

### Functional Enrichment Analysis of Co-Expression Module

To explore the function of genes in key co-expression modules, we uploaded the data to DAVID for analysis. DAVID is an online database (https://david.ncifcrf.gov/) (Tang et al., 2017; Nagy et al., 2018). It is a classic gene enrichment analysis website, mainly used for differential gene function and pathway enrichment analysis.

### Identification of Hub Genes In Key Module

After screening the key gene modules associated with the traits, the gene co-expression network map was drawn based on the relationships of the genes within the module. This network diagram belongs to the scale-free network. Mathematically, for a network graph, each node is given the concept of a degree, and the degree of a point refers to the number of edges associated with that point. In a scale-free network visualized by Cytoscape (3.7.0), the degree of a few nodes is significantly higher than the average point, and these points become hubs. A small number of hubs are associated with other nodes to form the entire network. The gene in the gene module that regulates the network center is the hub gene. At last, we decided the key genes through K-M survival analysis in the GEPIA database (http://gepia.cancer-pku.cn/).

### Validation of the Key Genes

We validated the function of candidate genes through a public databases, the ONCOMINE database (https://www.oncomine.org/) (Tang et al., 2017). Then, the overall survival and event-free survival analysis of hub genes were performed using Kaplan-Meier curve in Kaplan Meier plotter (https://kmplot.com/analysis/index.php?p=background) (Nagy et al., 2018) and LOGpc (Long-term Outcome and Gene Expression Profiling Database of pan-cancers) (http://bioinfo.henu.edu.cn/DatabaseList.jsp) (Wang et al., 2019). Finally, we performed multi-factor COX analysis on three key genes, established a risk model, and performed survival analysis and model identification.

## Results

### Data Preprocessing

**Figure 1:**
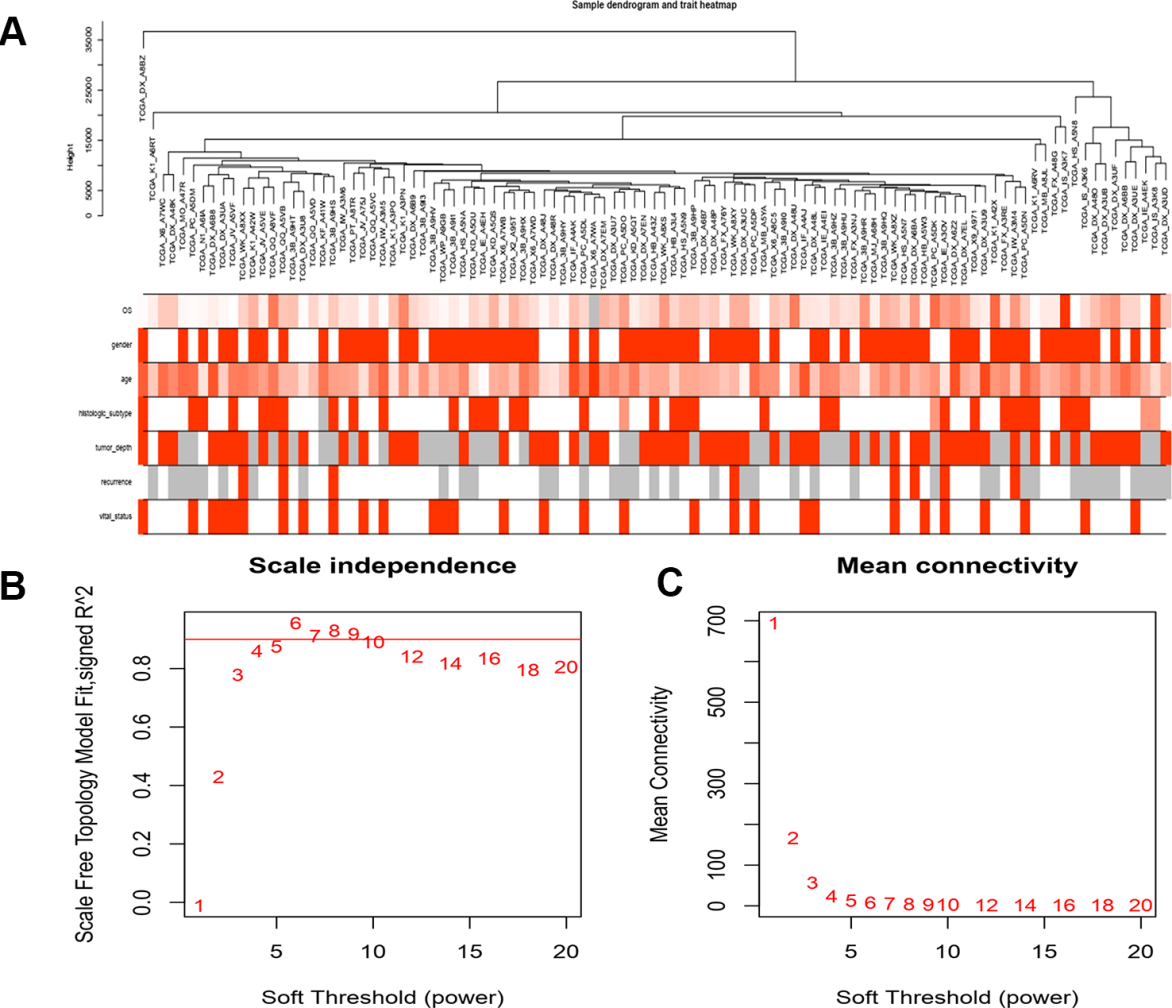
Clustering of samples and determination of soft-thresholding power. **(A)** The clustering was based on the expression data of LMS, which contained 103 LMS tumor tissue. The top 25% genes with the highest SD values were used for the analysis by WGCNA. **(B)** Analysis if the scale free fit index for various softthresholding powers (β). **(C)** Analysis of the mean connectivity of various soft-thresholding powers. In all, 4 was the most fit power value.

Gene annotation of gene expression data obtained from TCGA, matching probes and genes, removal of probes matching multiple genes, and gene annotation of the genes were matched by multiple probes using the median value as the final expression value. A total of 20,098 genes were identified. We calculated the variance of each gene and then selected the top 25% (5,025) of genes with the largest variance for WGCNA and sample cluster analysis (**Figure 1**).

### Construction of Co-Expression Modules

**Figure 2:**
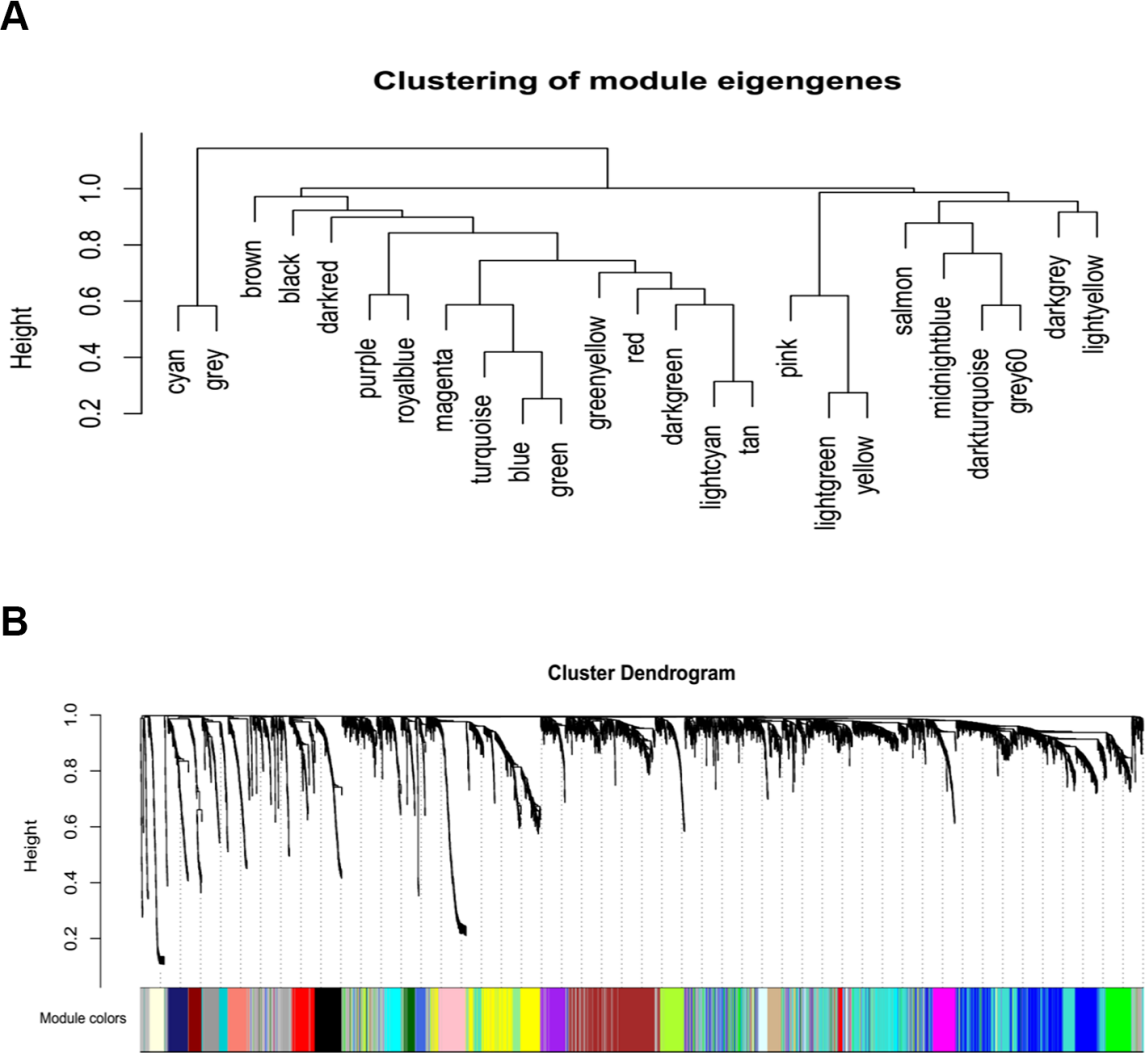
Construction of co-expression modules by WGCNA package in R. **(A)** The cluster dendrogram of module eigengenes. **(B)** The cluster dendrogram of genes. Each branch in the figure represents one gene, and every color below represents one co-expression module.

A gene co-expression network was constructed using weighted expression correlation. The soft threshold power value was used for initial screening. When the soft threshold power was equal to 4, the degree of independence reached 0.9 and the average connectivity was higher (**Figure 1**). Therefore, based on the weighted correlation, the WGCNA package automatically constructed a co-expression network, performed hierarchical clustering analysis, and segmented the clustered results according to the predetermined thresholds to obtain different gene modules. Of all the genes in the LMS network, 4,255 were assigned to 24 modules (**Table 1**), and the remaining 770 genes were assigned to the same “gray” module (**Figure 2**) and were included in the heat map. Branches of cluster trees and different color represent different clustering modules.

**Table 1:** Co-expressions modules.

Module color	Genes
Black	144
Blue	559
Brown	412
Cyan	112
Dark green	63
Dark gray	45
Dark red	68
Dark turquoise	58
Green	221
Green yellow	124
Gray	770
Gray 60	89
Light cyan	93
Light green	82
Light yellow	74
Magenta	132
Midnight blue	105
Pink	134
Purple	131
Red	151
Royal blue	68
Salmon	113
Tan	115
Turquoise	816
Yellow	346

### Correlation Between Modules and Identification of Key Modules

**Figure 3:**
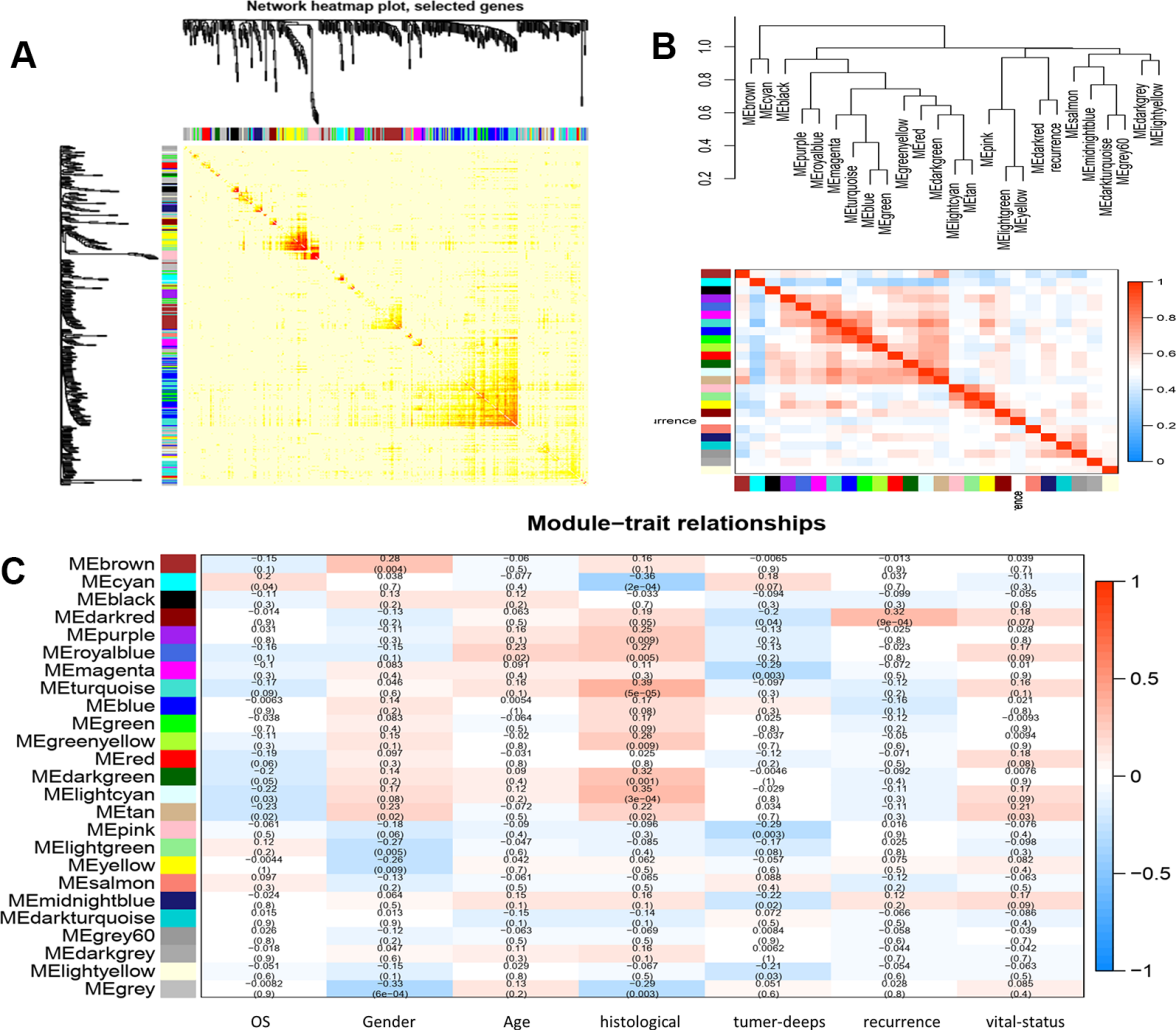
**(A)** interaction relationship analysis of co-expression genes. Different colors of horizontal axis and vertical axis represent different modules. The brightness of yellow in the middle represents the degree of connectivity of different modules. There was no significant difference in interactions among different modules, indicating a high-scale independence degree among these modules. **(B)** hierarchical clustering of module hub genes that summarize the modules yielded in the clustering analysis and heat map plot of the adjacencies in the hub gene network. **(C)** heat map of the correlation between module eigengenes and the clinical traits of LMS. The dark red module was the most positively correlated with recurrence of disease.

**Figure 4:**
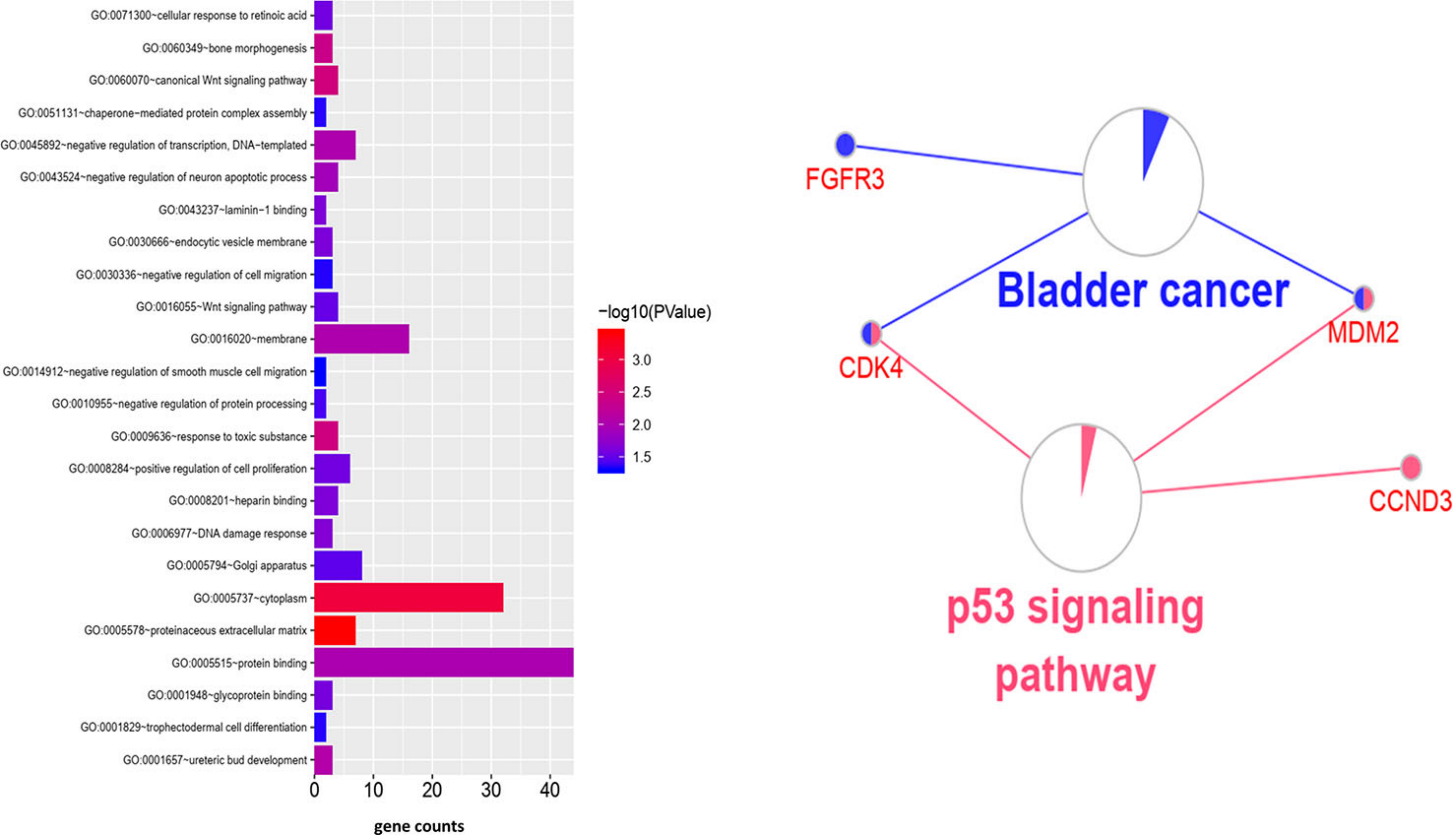
Go and KEGG enrichment analysis of genes in dark red module.

We calculated the eigengenes in-modules connectivity and clustered them to study the co-expression relationships of all modules. The results showed that each module was independent of the others, demonstrating the high degree of independence between modules and the relative independence of gene expression in each module. A heat map drawn from adjacent relationships showed similar results. The dark module ME was highly correlated with recurrence compared to other modules, suggesting that the dark module may play a key role in disease recurrence (**Figures 3** and **4**).

### Function Enrichment Analysis

To clarify the gene functions in the modules, we performed gene ontology enrichment analysis of the identified genes using DAVID, and explored combination of genes related to biological processes (BP), molecular functions (MF), and cellular components (CC) in key modules. (Details of GO enrichment are given in **Table 2**). GO analysis showed that these genes are involved in the components of the cell, embryo development, and transcription, and play an important role in the biological processes of cell division, signal transduction, and transcriptional regulation. The result of functional enrichment analysis showed that genes associated with biology processes were mainly enriched in GO:0060070 (canonical Wnt signaling pathway), GO:0009636 (response to toxic substance), GO:0060349 (bone morphogenesis), GO:0001657 (ureteric bud development), GO:0045892 (negative regulation of transcription, DNA-templated). Genes associated with Molecular Function were enriched in in GO:0043237 (laminin-1 binding), GO:0008201 (heparin binding), GO:0001948 (glycoprotein binding), GO:0005578 (proteinaceous extracellular matrix), and GO:0005737 (cytoplasm). According to the Kyoto Gene and Genomic Encyclopedia (KEGG) pathway analysis, the dark red module genes were mainly enriched in the p53 signaling pathway and the bladder cancer signaling pathway (**Table 3** and **Figure 4**). Practical ClueGo was used for visual analysis of KEGG pathway (Bindea et al., 2013).

**Table 2:** GO enrichment analysis of genes in co-expression modules.

Category	ID	Term	Count	P value
BP	GO:0060070	Canonical Wnt signaling pathway	4	3.4092E-03
BP	GO:0009636	Response to toxic substance	4	3.6460E-03
BP	GO:0060349	Bone morphogenesis	3	4.2975E-03
BP	GO:0001657	Ureteric bud development	3	8.3898E-03
BP	GO:0045892	Negative regulation of transcription, DNA-templated	7	9.3260E-03
BP	GO:0043524	Negative regulation of neuron apoptotic process	4	1.2264E-02
BP	GO:0006977	DNA damage response	3	2.1347E-02
BP	GO:0071300	Cellular response to retinoic acid	3	2.6763E-02
BP	GO:0008284	Positive regulation of cell proliferation	6	2.6878E-02
BP	GO:0016055	Wnt signaling pathway	4	3.0529E-02
BP	GO:0010955	Negative regulation of protein processing	2	3.9253E-02
BP	GO:0001829	Trophectodermal cell differentiation	2	4.6225E-02
BP	GO:0051131	Chaperone-mediated protein complex assembly	2	4.6225E-02
BP	GO:0030336	Negative regulation of cell migration	3	4.6720E-02
BP	GO:0014912	Negative regulation of smooth muscle cell migration	2	4.9693E-02
CC	GO:0005578	proteinaceous extracellular matrix	7	3.8200E-04
CC	GO:0005737	Cytoplasm	32	8.7000E-04
CC	GO:0016020	Membrane	16	9.3874E-03
CC	GO:0030666	Endocytic vesicle membrane	3	2.3207E-02
CC	GO:0005794	Golgi apparatus	8	3.3526E-02
MF	GO:0005515	Protein binding	44	1.0017E-02
MF	GO:0043237	Laminin-1 binding	2	2.2536E-02
MF	GO:0008201	heparin binding	4	2.2845E-02
MF	GO:0001948	Glycoprotein binding	3	2.5251E-02

**Table 3:** KEGG analysis of genes in co-expression modules.

Term	Count	P value	Genes		
hsa05219: Bladder cancer	3	0.009534445	FGFR3, MDM2, CDK4		
hsa05200: Pathways in cancer	6	0.01219952	WNT10B, FGFR3, BIRC7, MDM2, WNT11, CDK4		
hsa04550: Signaling pathways regulating pluripotency of stem cells	4	0.013674563	WNT10B, FGFR3, WNT11, KLF4		
hsa04115: p53 signaling pathway	3	0.02427143	CCND3, MDM2, CDK4		
hsa05205: Proteoglycans in cancer	4	0.034788315	WNT10B, SDC1, MDM2, WNT11		

### Identification of Hub Genes

**Figure 5:**
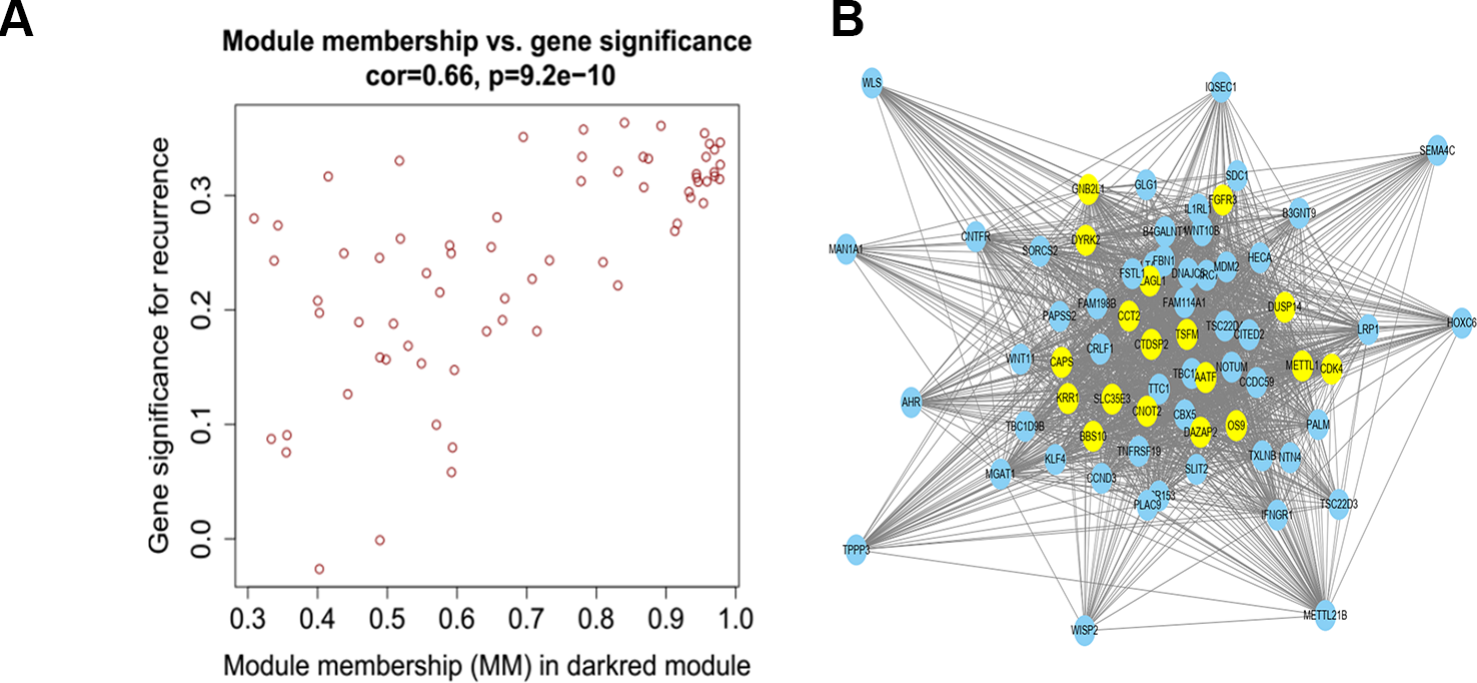
**(A)** Scatter plot of module eigengenes in the dark red module. **(B)** The hub genes in the dark red module and node size is correlated with connectivity of the gene by degree. Hubgene is represented as a bright yellow node in **(B)**.

**Figure 6:**
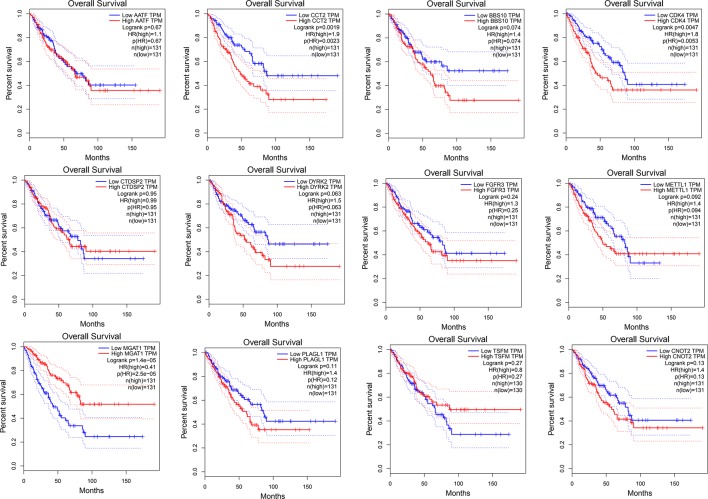
Survival analysis of 12 hub genes identified by WGCNA.

We used Cytoscape software to visualize the dark red module network to build the module and calculate connectivity within the module (Shannon et al., 2003). Genes with high connectivity were identified as hub genes based on connectivity within the module. Genes with significant survival results were selected and sorted by node degree (**Figure 5**). Twelve genes in the selected modules were considered hub genes: TSFM, AATF, BBS10, CDK4, CTDSP2, PLAGL1, DYRK2, FGFR3, CNOT2, METTL1, CCT2, and MGAT1. These hub genes were selected using cytoHubba (Chin et al., 2014). We used GEPIA (http://gepia.cancer-pku.cn/) to perform survival analysis on these hub genes to determine their biological significance (Tang et al., 2017). GEPIA was used to verify the expression characteristics of the twelve genes selected. Among these genes, CDK4, CCT2, and MGAT1 were associated with overall survival and recurrence-free survival, and the expression levels of these three genes were significantly higher in tumor tissues (**Figure 6**). Therefore, these genes were identified as key genes.

### Validation of Key Genes

**Figure 7:**
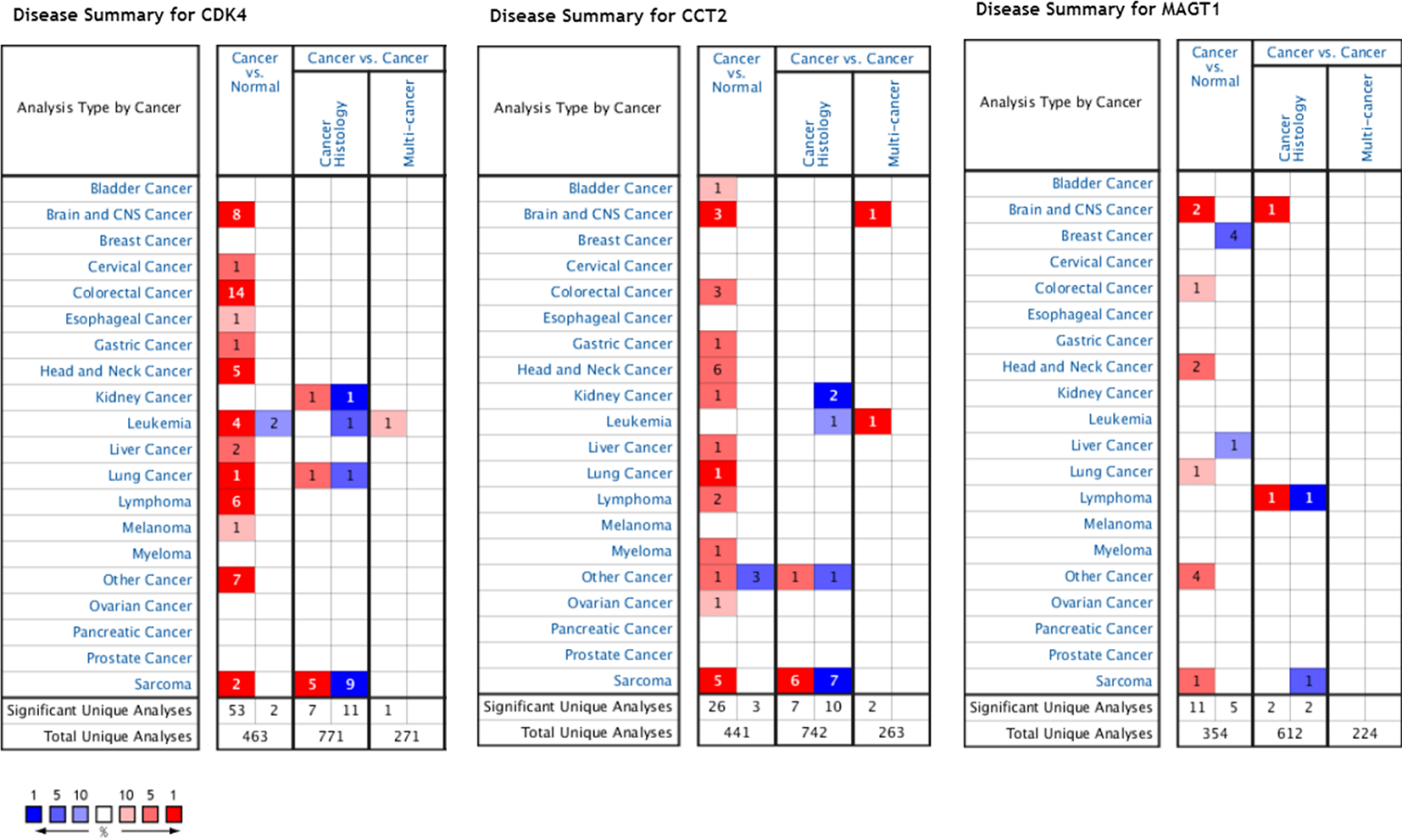
Expression profiles and analysis of cancer vs. normal tissue for CDK4, CCT2, and MAGT1 in human cancers analyzed using Oncomine.

**Figure 8:**
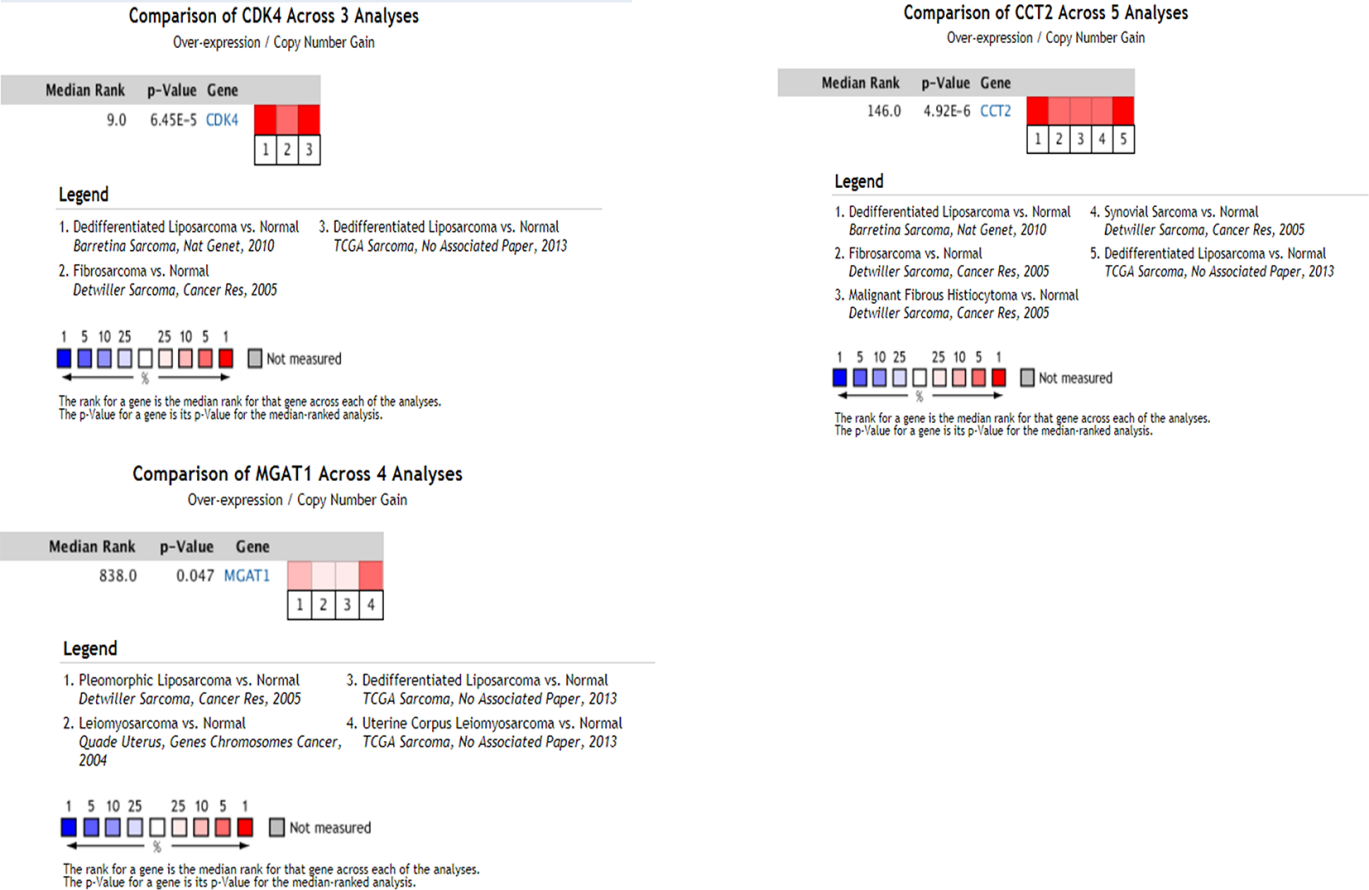
Oncomine analysis of cancer vs. normal tissue of CDK4,CCT2, and MAGT1. Heat maps of CDK4, CCT2, and MGAT1 gene expression in clinical sarcoma samples vs. normal tissues.

**Figure 9:**
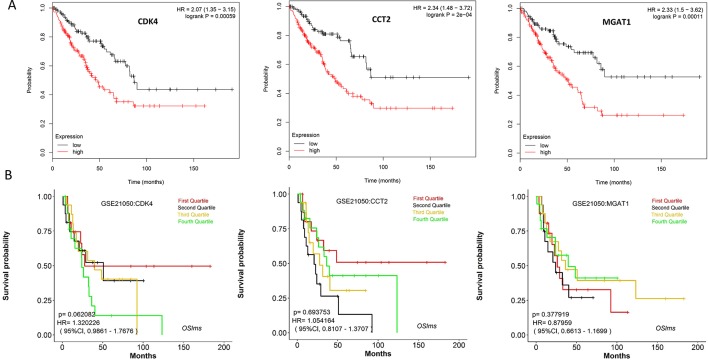
**(A)** Overall survival analyses of key genes were performed using Kaplan-Meier plotter **(B)** Event-free survival of key genes were performed using LOGpc. (P < 0.05 was considered statistically significant.)

**Figure 10:**
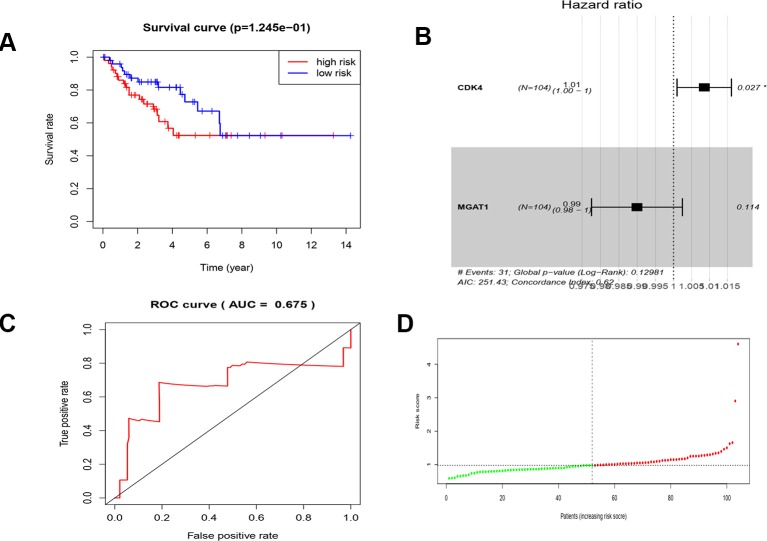
COX analysis of the key genes. **(A)** Survival analysis of high risk and low risk. **(B)** The hazard ratio of key genes of CDK4 and MGAT1. **(C)** ROC curve and AUC value. **(D)** Risk score of the patients.

Using the data from ONCOMINE database (https://www.oncomine.org/), we noted that leiomyosarcoma patients who had an association of genomic alterations in CDK4,CCT2, and MGAT1 (**Figure 7**). The expression of the three key genes in the dark red module positively correlated with the disease state. Oncomine analysis of cancer vs. normal tissue showed that cdk4, cct2, and MGAT1 were significantly overexpressed in leiomyosarcoma in the different datasets (**Figure 8**) (Quade et al., 2004; Detwiller et al., 2005; Nakayama et al., 2007; Barretina et al., 2010; Chibon et al., 2010). Using the data from Kaplan Meier plotter and LOGpc, we noted that leiomyosarcoma patients who had an association of genomic alterations in CDK4, CCT2, and MGAT1 showed reductions in overall and disease-free survival. However, those observations were statistically significant for overall survival time and no statistically significant for event-free survival (**Figure 9**). Finally, through multi-factor COX analysis of the three genes, we obtained the risk prediction formula, risk score = CDK4* 0.00848 + MAGT1*(−0.01012) (**Table 4**). The model is analyzed for survival and the ROC value is calculated for verification (**Figure 10**).

**Table 4:** COX analysis of key genes.

id	Coef	HR	HR.95L	HR.95H	P value
CDK4	0.008487993	1.008524118	1.000962917	1.016142437	0.027061684
MGAT1	–0.010120172	0.989930865	0.977568773	1.002449286	0.114468861

## Discussion

Leiomyosarcoma, which occurs in smooth muscle connective tissue, accounts for ten percent of all soft tissue sarcomas. LMS is malignant and exhibits a high degree of invasiveness, high recurrence rate, and high mortality (George et al., 2018). LMS can occur in any location of the body such as in the extremities, small intestine, or retroperitoneal space. As LMS is most common in the uterus, it can be classified as uterine LMS (ULMS) or non-uterine LMS (NULMS) (Guo et al., 2015). ULMS is highly aggressive and is not sensitive to chemotherapy and radiation therapy. Surgical resection is currently the best treatment. The median survival of individuals with NULMS and ULMS is less than 5 years (Eriksson, 2010). In-depth study of the biological behavior and potential molecular mechanisms of LMS is of great significance for improving the efficacy and prognosis of LMS (Pautier et al., 2015; Schoffski et al., 2016; Mir et al., 2016; Tawbi et al., 2017; Gronchi et al., 2017).

In this study, we assessed gene expression to identify potential biomarkers for LMS using WGCNA. Twenty-four co-expression modules were constructed for 5,025 genes from 103 human LMS samples. Because WGCNA focuses on the association between co-expression modules and clinical features, the results are more reliable and biologically meaningful. Genes that are functionally related to each other are clustered together in the same module. Thus, WGCNA can identify biologically relevant modules and central genes that can ultimately become biomarkers for detection or treatment. We found that the dark red module was most significantly associated with disease recurrence. Using a total of 68 genes in the dark red module were screened. GO and KEGG analyses showed that these genes are involved in the components of the cell, embryo development, and transcription, and play an important role in the biological processes of cell division, signal transduction, and transcriptional regulation. We believe that the dark red module was the most important module for characterization of the LMS recurrence mechanism.

Further analysis of the dark red module showed that three genes (CDK4, CCT2, and MGAT1) significantly correlated with survival analysis and were identified as hub genes. The hub genes were further validated in GEPIA and ONCOMINE. Some studies have reported that these three key genes are cancer-associated genes involved in mitotic regulation in cancer cells and inhibition of cell proliferation, which may contribute to tumorigenesis and malignant phenotype. CDK4 is an important effector of the P53 signaling pathway. CDK4 encodes a member of the Ser/Thr protein kinase family, which is important for cell cycle G1 progression. Mutations of this gene and its related proteins, including D-type cyclins, p16 (INK4a), and retinoblastoma gene product (Rb), have been found to be involved in tumorigenesis in a variety of cancers. Multiple polyadenylation sites of this gene have been reported (O'Leary et al., 2016). Increased expression of CDK4 is associated with advanced soft tissue sarcomas and is often observed in many types of cancer. This may be due to an imbalance in the cyclin D-CDK4/6-INK4-Rb pathway, leading increased abnormal cell proliferation (Lucchesi et al., 2018). The expression of CDK4 in tumor tissues is specific and can provide a sensitive marker for diagnosis of low-grade osteosarcoma (Dujardin et al., 2011). Targeted therapy has recently received increased attention. Inhibitors of CDK4/6 have been shown to have significant activity against several solid tumors, increase intracellular double-stranded RNA levels, and activate endogenous retroviral elements to inhibit tumor cell expression (Goel et al., 2017). Due to the importance of CDK4/6 activity in tumorigenesis, targeted inhibitors of the CDK4/6 gene have become new candidates for tumor therapy. The CDK4 inhibitor letrozole has been used to successfully treat breast cancer and has recently entered clinical trials for treatment of various diseases (Klein et al., 2018). CCT2 is a member of a chaperone protein containing the TCP1 complex (CCT), also known as the TCP1 loop complex (TRiC). It is a macromolecular complex of 16 subunits forming a back-to-back bicyclic structure, each ring containing eight different subunits α, β, γ, δ, ϵ, ζ, η, and θ (CCT1–8). This gene has been found to encode two different transcript variants. CCT has the function of assisting the correct folding of proteins, and cytoskeletal proteins and cell cycle regulators are the most important substrates. Blocking CCT activity can cause significant morphological changes and cell cycle arrest. Previous studies have found that CT2 is overexpressed in some tumors, and CCT2 expression in intestinal and hepatocarcinoma tissues is significantly higher than that in adjacent tissues, and its expression is highly correlated with PCNA, suggesting that CCT2 may be involved in cell proliferation. The positive expression of CCT2 in gallbladder carcinoma is associated with TNM stage and lymph node metastasis. In addition, the expression of CCT2 is associated with histological grade, suggesting that it is associated with tumor differentiation and progression. Recent studies have found that CCT2 is critical for the survival of breast cancer patients, and is significantly higher in hepatocellular carcinoma, colon cancer, extrahepatic cholangiocarcinoma, gallbladder cancer, and gastric cancer than benign lesions and normal tissues. However, how CCT2 affects HCC proliferation and progression remains to be explored. In conclusion, CCT2 is closely related to the development of HCC, which provides a theoretical basis for CCT2 to become a target for HCC molecular targeted therapy (Amit et al., 2010; Zou et al., 2013; Guest et al., 2015; Pavel et al., 2016; Minegishi et al., 2018). In our study, positive expression of CCT2 was negatively correlated with survival time, and was an independent risk factor for prognosis of LMS. The main function of monoacylglycerol acyltransferase (MGAT) is to catalyze the synthesis of diacylglycerol by monoacylglycerol. Currently, three genes encoding MGAT have been found, namely MGAT1, MGAT2, and MGAT3. MGAT is an important gene for the synthesis of diacylglycerol during fat deposition, and is closely related to the absorption of fat in the intestine, the synthesis and storage of lipids, and intracellular signal transduction. An important function of MGAT1 is an important target of the Wnt/β-catenin signal. The protein has a characteristic type II transmembrane protein characteristic and plays an important role in the early development of animal embryos, organ formation, tissue regeneration, and other physiological processes, and is considered to be essential for normal embryogenesis. Stable overexpression of the MGAT1 gene in the Huh7 cell line resulted in a significant increase in tumor growth rate in severe combined immunodeficiency (SCID) mice. Down-regulation of MGAT1 expression in the liver can significantly reduce hepatic steatosis in mice, while reducing body weight and increasing glucose tolerance (Lee et al., 2012; Akiva and Birgul Iyison, 2018).

The results showed that the expression of CDK4, CCT2, and MGAT1 in LMS tissues was significantly higher than that in adjacent tissues and an important member of the cancer signaling pathway. Clinical data from the GEPIA dataset confirms that CDK4, CCT2, and MGAT1 expression levels are highly correlated with prognosis, and that up-regulation may lead to a significant reduction in survival time in patients with soft tissue sarcoma. At last, the result of cox analysis suggests that CDK4 and MGAT1 may play an important role in the development of LMS and can be used as predictors of LMS patients as a post-evaluation indicator. A recent large cohort study of 99 patients with LMS found that CDK4 may be a key gene for leiomyosarcoma recurrence, and palbociclib, an inhibitor of CDK4, may provide a new option for targeted therapy in patients with LMS (Bohm et al., 2019). However, LMS tumorigenesis is not well understood, and further evaluation of large sample clinical data is critical.

We studied co-expressed gene modules that were highly correlated with tumor recurrence, and determination of hub genes in these module helped to determine the major functions of the genes in these modules. The study of these three central genes may help us to understand the molecular mechanisms of tumorigenesis and these genes may represents new diagnostic marker and therapeutic target for LMS.

## Data Availability Statement

Publicly available datasets were analyzed in this study. This data can be found here: https://tcga-data.nci.nih.gov/tcga/.

## Author Contributions

JY and CL reviewed relevant literature and drafted the manuscript. JZ, XL and SW conducted all statistical analyses. JY and SW were responsible for the supervision of the project and final approval of the version. All authors read and approved the final manuscript.

## Conflict of Interest

The authors declare that the research was conducted in the absence of any commercial or financial relationships that could be construed as a potential conflict of interest.
